# Shark Fin Electrocardiogram: A Deadly Electrocardiogram Pattern in Takotsubo Cardiomyopathy During 15 Years of Follow-Up

**DOI:** 10.7759/cureus.36509

**Published:** 2023-03-22

**Authors:** Atheer M Almutairi, Wed S Alotaibi, Alanoud H Almuhana, Ihab Suliman

**Affiliations:** 1 College of Medicine, King Saud Bin Abdulaziz University for Health Sciences College of Medicine, Riyadh, SAU; 2 Cardiology, King Abdulaziz Medical City, King Abdulaziz Cardiac Center, Ministry of National Guard Health Affairs, Riyadh, SAU

**Keywords:** st-segment elevation myocardial infarction (stemi), cardiac stressor, hypokinesis, takotsubo cardiomyopathy (ttc), shark fin

## Abstract

Takotsubo cardiomyopathy (TTC) or stress-induced cardiomyopathy is characterized by transient left ventricular apical ballooning in the absence of coronary occlusion. Although the underlying mechanism is still unknown, exaggerated sympathetic nervous system and catecholamine cardiotoxicity, followed by metabolic disturbance, and multi-vessel epicardial coronary artery vasospasm, are thought to be responsible for the development of this condition. TTC accounts for 1-2% of patients presenting with the acute coronary syndrome (ACS) with the majority of patients being postmenopausal women. Shark fin electrocardiogram (SFE) or triangular ST-segment elevation is an uncommon electrocardiogram (ECG) finding that is typically associated with an increased risk of ventricular fibrillation and cardiogenic shock, thus, it is considered a poor prognostic factor. We present a case of a 57-year-old postmenopausal female with TTC post-colonic perforation. Upon further investigation, an ECG revealed an SFE or triangular ST-segment elevation on the anterolateral leads, and an elevated serum troponin level was found. On trans-thoracic echocardiogram (TTE), hypokinesis and akinesis of the apex and left ventricular segments were observed with sparing of the basal segments. Eventually, the patient was successfully managed and monitored until regain of normal function.

## Introduction

Takotsubo cardiomyopathy (TTC), also known as an apical ballooning syndrome or stress cardiomyopathy, is characterized by transient left ventricular apical ballooning in the absence of coronary occlusion [1]. This condition is triggered by intense emotional stressors and/or physical stressors, including neurological disorders, sepsis, and pheochromocytoma [2]. Although the exact pathophysiology is still unknown, an exaggerated sympathetic nervous system-induced myocardial weakness is thought to be the main precipitating mechanism of this condition [3]. Another theory for TTC is that dysregulated action of the catecholamines in the cardiac tissue was found to be associated with corticosteroid hormone deficiency [4]. The excessive catecholamine release in TTC causes cardiotoxicity and multi-vessel spasms resulting in transient myocardial stunning [3]. TTC is prevalent in 1-2% of acute coronary syndrome (ACS) patients, with the majority of patients being postmenopausal women [5].

Clinical features of TTC usually overlap with those seen in ACS patients and are characteristically preceded by stressful events [6]. Symptoms predominately include retrosternal chest pain that mimics angina, dyspnea, syncope, arrhythmias, signs of heart failure, and/or signs of cardiogenic shock such as hypotension and pulmonary edema. The cardiac enzymes and biomarker levels are mildly elevated in TTC [6,7]. Additionally, on electrocardiogram (ECG), T wave inversion and/or low amplitude ST-segment elevation in the precordial leads are the most common abnormalities observed [6,7]. Thus, it is difficult to differentiate between TTC and ACS based on ECG and laboratory findings alone. In a retrospective study of 33 patients with TTC, authors proposed ECG criteria to differentiate TTC from anterior myocardial infarction. Absence of aberrant Q waves, reciprocal alterations, ST-segment elevation in lead V1, and the presence of ST-segment elevation in lead aVR provided 91% sensitivity and 96% specificity for TTC [7]. Moreover, triangular ST-segment elevation or lambda-wave ST-elevation is an alarming ECG pattern usually associated with ST-elevation myocardial infarction (STEMI). It has been reported in rare cases of TTC and is usually seen in critically ill patients with septic shock [8].

The key feature of acute TTC on two-dimensional trans-thoracic echocardiogram (TTE) is apical ballooning and the relative compensatory hypercontractility of the basal segments. An angiography is crucial for the diagnosis of TTC. However, in the intensive care unit (ICU), angiography might not be obtainable in severe, unstable cases [9]. Therefore, clinical judgment is sufficient for diagnosis in the ICU [9]. The following is a case of a postmenopausal female with a shark fin electrocardiogram (SFE)-associated TTC post-colonic perforation. Complete resolution and regain of normal function were evident on TTE and ECG during 15 years of follow-up.

## Case presentation

**Figure 1:**
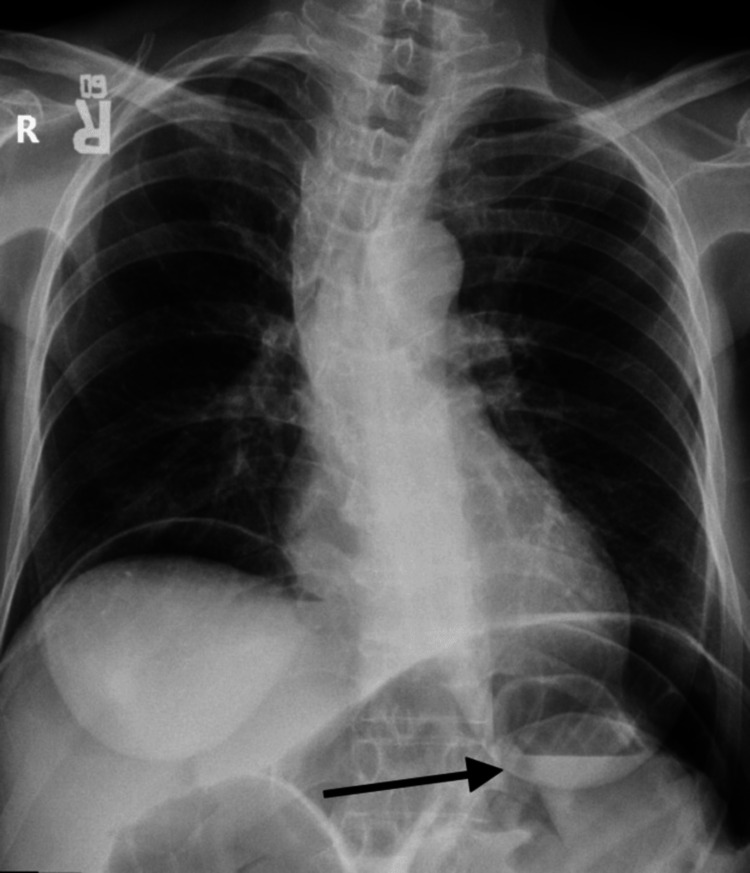
Chest X-ray Free air was noted in the peritoneal cavity.

A 57-year-old postmenopausal female with a past medical history significant for colorectal carcinoma status post resection in 2007. She was on chemotherapy for two years post-resection. On 27/12/2008, she complained of sudden onset of intense stabbing pain for one day followed by diffuse abdominal pain and distention associated with vomiting and fever. The pain radiated to the back and was partially relieved by analgesia. There was no history of change in bowel habits, rectal bleeding, and urinary symptoms. The systemic review was unremarkable. Upon examination, the patient looked ill and had tachycardia with a pulse rate of 122 beats/minute. The patient was hypotensive with a blood pressure of 95/67 mmHg. The respiratory rate was 20 breaths/minute, and the oxygen saturation was 95%. Additionally, the chest was clear and the cardiovascular examinations were unremarkable. Abdominal examination was normal except for scars from the previous operation with generalized tenderness and guarding upon palpation and decreased bowel sounds on auscultation. Laboratory tests were obtained, and a complete blood count (CBC) showed a white blood cell (WBC) count of more than 11.000 WBCs per microliter which indicated leukocytosis, and hemoglobin level was 13 grams per deciliter which were normal. The metabolic panel showed a low sodium level of 129 milliequivalents per liter. The liver function test was normal. Chest x-ray showed air in the diaphragm and a distended abdomen with multi-air-fluid levels (Figure 1).

According to X-ray findings, bowel perforation was suspected and the patient was taken to the operation room (OR) for exploratory laparotomy, which showed adhesive intestinal obstruction and a bowel perforation. The patient was eventually managed with small bowel resection and primary anastomosis. Postoperatively, she was admitted to the ICU where she was intubated and sedated. During her ICU admission, the patient’s clinical status deteriorated. Despite the fluid resuscitation, she was in severe shock and hypotensive. Inotropes and broad-spectrum antibiotics were given. Six hours later, the patient was improving and weaning off inotropes. Laboratory workup during the ICU stay showed mildly elevated troponin, thus, she was followed by a cardiologist during the postoperative period. TTE showed a severely reduced systolic function with an estimated left ventricular ejection fraction of less than 25%. Additionally, severe global hypokinesis to akinesis of the left ventricle was evident on TTE where only the basal segments contracted well. The right ventricle and left atrial were normal in size and function (Video 1).

**Video 1:** Echocardiogram 1 End diastolic volume (EDV): 66.6 mL End systolic volume (ESV): 17.1 mL Ejection fraction: <25% Left atrial dimension: 3.0 cm

**Figure 2:**
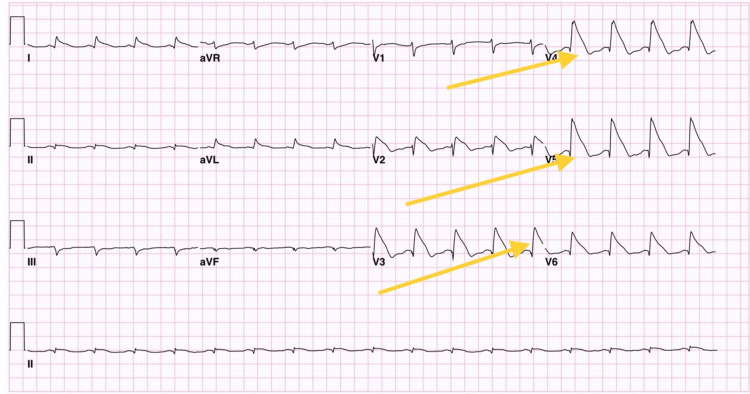
Electrocardiogram Electrocardiogram showed a shark fin pattern on the anterolateral leads.

An ECG was done and showed an SFE on the anterolateral leads (Figure 2).

Considering her clinical picture, cardiac catheterization was strongly indicated, however, it was not performed due to her clinical status. She was declared hemodynamically unstable and her family refused to do the procedure. Eventually, the most likely diagnosis was TTC post colonic perforation due to recovery of left ventricular function, normalization of the ECG, and drop of cardiac enzymes within one week after management. During that period, she was treated conservatively and medically with inotropes. After weaning of inotropes, she was on angiotensin-converting enzyme (ACE) inhibitors and beta-blockers (BB) until discharge. A year before the crisis in 2007, a TTE was performed (Video 2). The left ventricular systolic function was normal with an LVEF of more than 55%. No regional wall motion abnormalities were found and the left ventricle was hyper-dynamic. The trans-mitral spectral Doppler flow pattern was suggestive of impaired left ventricular relaxation.

**Video 2:** Echocardiogram 2 End diastolic volume (EDV): 8 mL Left atrial dimension: 3.3 cm Ejection fraction: >55%

**Figure 3:**
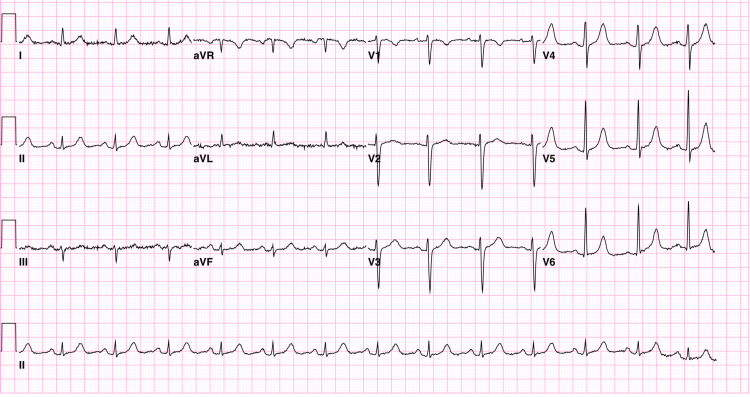
Electrocardiogram The electrocardiogram showed normal sinus rhythm with right atrial enlargement.

 The ECG showed a normal sinus rhythm with a right atrial enlargement (Figure 3).

Six years after the crisis in 2014, a TTE revealed a normal left ventricular systolic function with an LVEF of more than 55% (Video 3). The right ventricle was normal in size and function. The transmittal spectral Doppler flow pattern was suggestive of impaired left ventricular relaxation. The mitral and tricuspid valves were normal in structure and function with mild mitral and tricuspid regurgitation. The right ventricle systolic pressure was normal.

**Video 3:** Echocardiogram 3 End diastolic volume (EDV): 107.0 mL End systolic volume (ESV): 34.1 mL Left atrial dimension: 3.3 cm Ejection fraction: >55%

**Figure 4:**
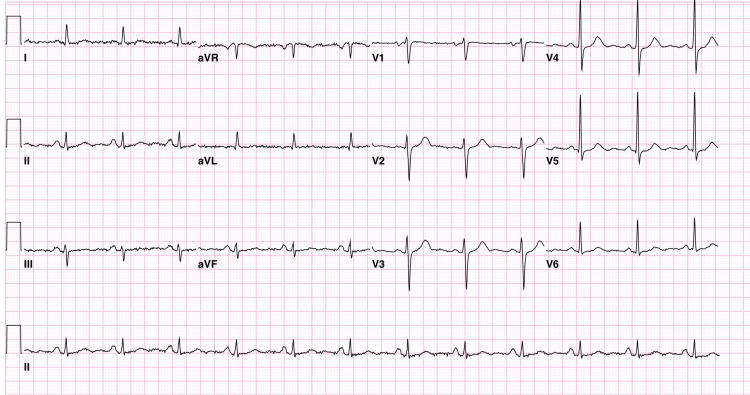
Electrocardiogram Electrocardiogram obtained on 01/02/2023, which showed normal sinus rhythm.

Fifteen years later on 1/2/2023, the patient was seen in the outpatient clinic vitally stable, with normal sinus rhythm on ECG (Figure 4).

## Discussion

TTC is a rare syndrome that is associated with the physical or extreme emotional stress that leads to rapid and severe reversible cardiac dysfunction. The exact cause is unknown but it is thought to be caused by a sudden excess of catecholamines released at the time of a stressful event inducing microvascular dysfunction [8]. The catecholamine-mediated effects on the heart result in structural changes such as increased extracellular matrix, contraction band necrosis, and mild neutrophilic infiltration. There are several potential causes, such as inadequate anesthesia depth, exogenous epinephrine injection, and anaphylaxis secondary to the release of inflammatory mediators (Kounis syndrome), which may contribute to the hyper-catecholaminergic condition during surgery [10]. Our patient may have been exposed to exogenous catecholamines through the injection of a local anesthetic mixed with epinephrine before beginning the procedure, which may have led to an increased catecholaminergic state and subsequent TTC. The patient's gender and age are additional important factors.

A recent review of a case series revealed that approximately 90% of all reported cases of TTC have occurred in postmenopausal females between the ages of 58 and 75 years. The reason for the female predominance is unknown but raises the question as to whether withdrawal from estrogens contributes to the pathogenesis [10]. A case was reported by Pfister et al. of a 31-year-old pre-menopausal woman who experienced an atypical presentation of stress-related cardiomyopathy after major breast surgery [11]. Although she did not exhibit the TTC features often seen on left ventriculography, she did present with other characteristics commonly found which included pulmonary edema, diffuse T-wave abnormality, severe left ventricular dysfunction, and elevated troponin levels. The authors assume that prolonged surgery with catecholamine release may have contributed to the development of left ventricular dysfunction along with postoperative pain, length of surgery, anesthesia withdrawal, aggressive breast cancer, or intravascular fluid volume shifts [11].

In TTC, ACS is the primary differential diagnosis suspected. Therefore, an ECG is an initial investigation obtained [12]. In comparison with STEMI, the amplitude of the ST segment was much lower in patients with TTC. In 15% of TTC patients, a normal or nonspecific ECG result is frequently observed. As the ST segments normalize, diffuse T-wave inversions tend to occur [13]. TTC cannot be accurately distinguished from STEMI based on ECG results only. SFE should be differentiated from other conditions causing similar waveforms such as wide-complex tachycardia and hyperkalemia [14]. In a retrospective study of 33 patients with TTC, authors proposed ECG criteria to differentiate TTC from anterior myocardial infarction. On ECG, the absence of aberrant Q waves, reciprocal alterations, ST-segment elevation in lead V1, and the presence of ST-segment elevation in lead aVR provided 91% sensitivity and 96% specificity for TTC [7]. SFE, also known as 'deadly ECG sign', 'lambda-wave', or 'giant R waves', is an ECG sign that is described as a complex phenomenon where there is a fusion of the QRS, ST, and the T waves. SFE is known to be a poor prognostic factor associated with cariogenic shock and ventricular fibrillation, thus it's known as the deadly ECG sign. SFE is commonly found to be an indicator of severe ACS and ischemic heart disease [15]. TTC is a non-ischemic cause of the SFE associated with the downsloping of the ST segment pattern. The presence of SFE along with TTC is rare and it is thought to be in a parallel relationship with the amount of stress leading to cardiogenic fatigue. Tarantino et al. described the SFE in five patients in a larger study that included 158 consecutive patients with TTC. Ventricular dysfunction, in-hospital complications, and long-term adverse outcomes were found connected in 80% of the cases [16]. According to the literature, there are only a few cases reported of TTC. It was reported that SFE was found in only 1.4% of STEMIs [17]. Patients with SFE will require aggressive management and monitoring in the ICU. Verdoia et al. reported a case of a 51 years old female that developed TTC post-sepsis in the ICU with the SFE sign. The patient regained complete sinus rhythm post-acute management and was subsequently followed up for a period of six months [18].

Patients with TTC are advised to have follow-ups with a cardiologist following diagnosis to assure remission of the cardiomyopathy, typically with serial echocardiograms. Thereafter, annual clinical follow-ups are advised. In TTC, the prognosis is often excellent, with over 95% of patients having full recovery between four and eight weeks after hospital admission [19]. According to a study by Singh et al., the annual recurrence rate is around 1.5%, and the mortality estimates have ranged between 1% and 3.2%. Twenty percent of cases of TTC experience complications, especially in the early stages. Common complications include the following: pulmonary edema, left-sided heart failure, cardiogenic shock, left ventricular outflow obstruction, mitral regurgitation, and death [20].

## Conclusions

SFE is an uncommon ECG finding that is typically associated with an increased risk of ventricular fibrillation and cardiogenic shock. The presence of SFE in TTC patients is a poor prognostic factor, thus, aggressive management and mechanical circulatory support are required. We present a case of TTC with triangular ST-segment elevation (shark fin sign) as consequent to colonic perforation. The patient had a complete recovery of normal function upon discharge and during 15 years of follow-up.
